# Efferocytosis and Its Associated Cytokines: A Light on Non-tumor and Tumor Diseases?

**DOI:** 10.1016/j.omto.2020.04.010

**Published:** 2020-04-28

**Authors:** Danfeng Lin, Xiaodiao Kang, Lu Shen, Sheng Tu, Cameron Lenahan, Yiding Chen, Xiaochen Wang, Anwen Shao

**Affiliations:** 1 Department of Surgical Oncology, The Second Affiliated Hospital, Zhejiang University School of Medicine, Hangzhou, China; 2 Department of Orthopedics Surgery, The Second Affiliated Hospital and Yuying Children’s Hospital of Wenzhou Medical University, Wenzhou, China; 3 State Key Laboratory for Diagnosis and Treatment of Infectious Diseases, Collaborative Innovation Center for Diagnosis and Treatment of Infectious Diseases, The First Affiliated Hospital, College of Medicine, Zhejiang University, Hangzhou, China; 4 Burrell College of Osteopathic Medicine, Las Cruces, NM, USA; 5 Center for Neuroscience Research, School of Medicine, Loma Linda University, CA, USA; 6 Department of Breast Surgery, Zhejiang Provincial People’s Hospital, People’s Hospital of Hangzhou Medical College, Hangzhou, China; 7 Department of Neurosurgery, The Second Affiliated Hospital, Zhejiang University School of Medicine, Hangzhou, China

**Keywords:** efferocytosis, inflammation, cytokine, atherosclerosis, lung disease, tumor, breast cancer, prostate cancer, leukemia, target

## Abstract

Billions of cells undergo turnover and die via apoptosis throughout our lifetime. A prompt clearance of these apoptotic cells and debris by phagocytic cells, a process known as efferocytosis, is important in maintaining tissue homeostasis. Accordingly, impaired efferocytosis due to the defective clearance and disrupted stages can lead to a growing number of inflammation- and immune-related diseases. Although numerous studies have shown the mechanisms of efferocytosis, its role in disorders, such as non-tumor and tumor diseases, remains poorly understood. This review summarizes the processes and signal molecules in efferocytosis, and efferocytosis-related functions in non-tumor (e.g., atherosclerosis, lung diseases) and tumor diseases (e.g., breast cancer, prostate cancer), as well as describes the role of involved cytokines. Of note, there is a dual role of efferocytosis in the abovementioned disorders, and a paradoxical effect among non-tumor and tumor diseases in terms of inflammation resolution, immune response, and disease progression. Briefly, intact efferocytosis and cytokines promote tissue repair, while they contribute to tumor progression via the tumor microenvironment and macrophage politzerization. Additionally, this review provides potential targets associated with TAM (TYRO3, AXL, MERTK) receptors and cytokines, such as tumor necrosis factor α and CXCL5, suggesting potential novel therapeutic ways in treating diseases.

## Graphical Abstract

**Figure:**
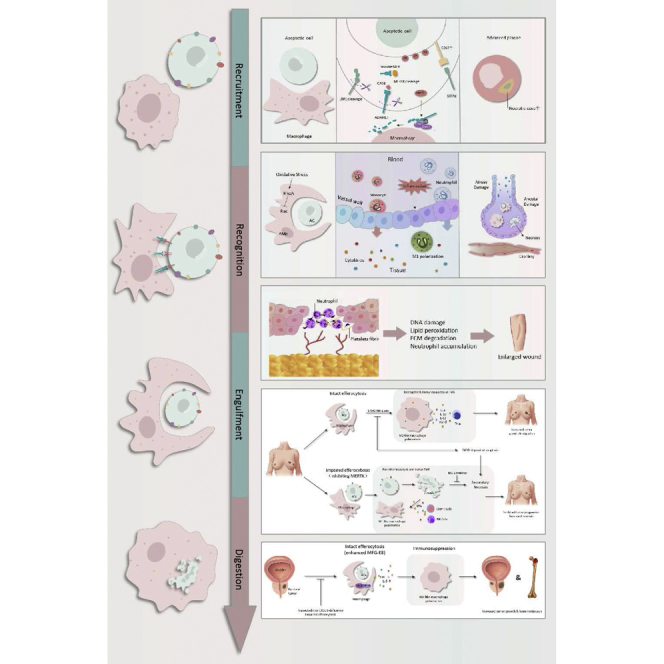

## Teaser

A clearance of apoptotic cells by phagocytic cells, a process known as efferocytosis, is important in maintaining tissue homeostasis. Impaired efferocytosis due to defective clearance leads to inflammation- and immune-related diseases. This review summarizes the processes and signal molecules in efferocytosis, and efferocytosis-related functions in non-tumor and tumor diseases, as well as describes the role of involved cytokines.

## Main Text

Nearly 200–300 billion cells undergo turnover, and 0.4% of the estimated 37.2 trillion cells in an adult die every day.^1^^,^^2^ During the homeostatic cell turnover, an immunologically non-inflammatory process, known as efferocytosis, is involved. Efferocytosis is a term derived from the Latin *efferre*, meaning to carry the dead to the grave. It describes the process by which apoptotic cells (ACs) are recognized and consumed by phagocytic cells, including professional phagocytes (e.g., macrophages and immature dendritic cells [DCs]) and nonprofessional phagocytes (e.g., epithelial and endothelial cells). Prompt clearance of ACs assists in preventing the leakage of intracellular contents that produce tissue inflammation, and to ensure that there is adequate space for young, healthy cells. Recent studies have identified the important role of efferocytosis in inflammation resolution, immune tolerance, cancer development, and tissue homeostasis. In addition, numerous studies have demonstrated that either intact or defective efferocytosis can have positive or negative effects on the body through various signals and released cytokines.^3^ For example, in tumors such as breast cancer, efferocytosis influences the tumor microenvironment (TME) and facilitates tumor growth, while impaired efferocytosis increases secondary necrosis.^4^ In brief, there is a dynamic balance of efferocytosis in the body, and a better understanding of efferocytosis may shed light on key pathophysiological processes.

### Overview of Efferocytosis Process and Signal Molecules

**Figure 1:**
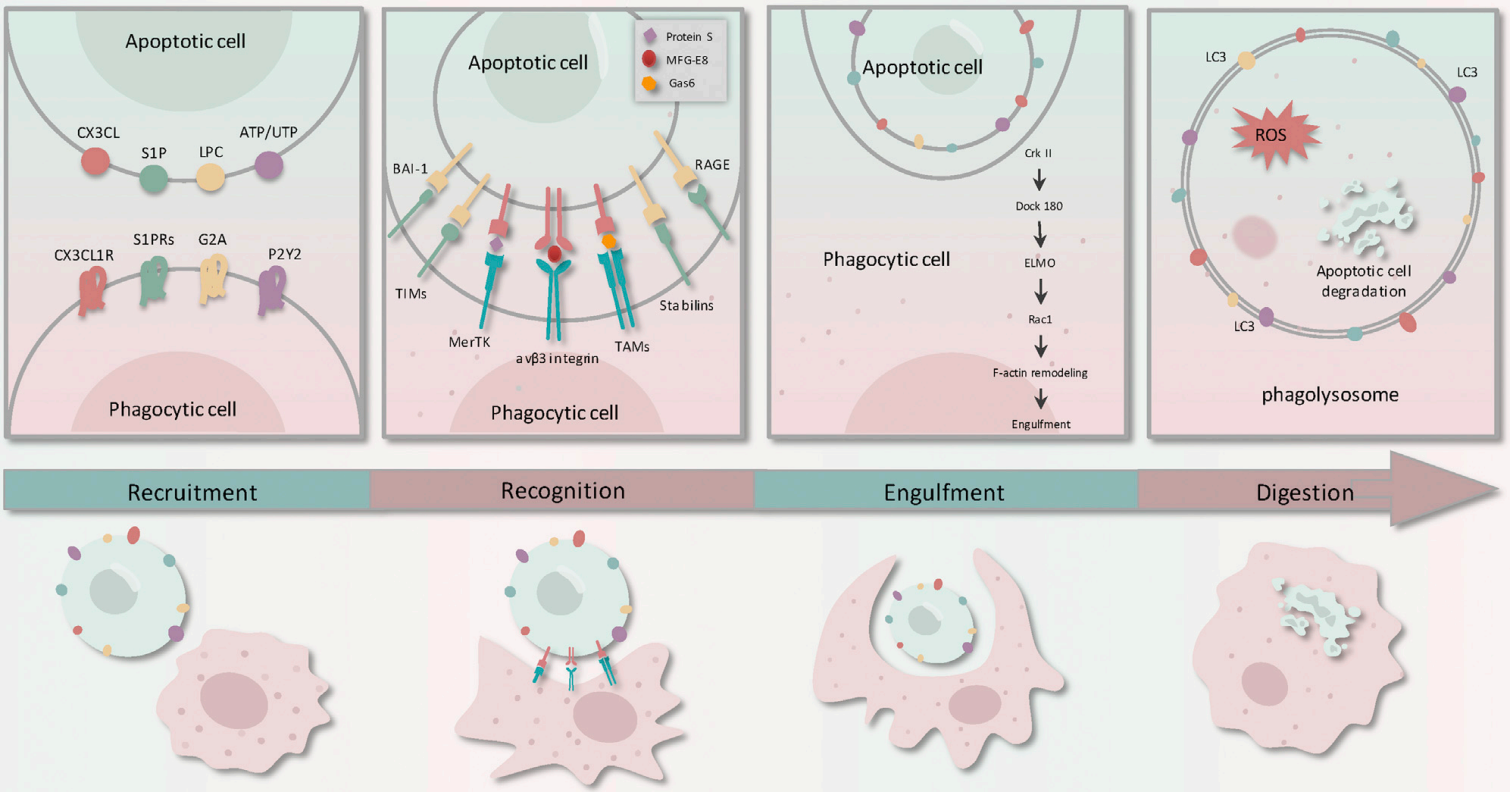
The Process and Mechanism of Efferocytosis The process of efferocytosis involves four steps, including the recruitment of phagocytic cells and the recognition, phagocytosis, and digestion of apoptotic cells.

The process of efferocytosis involves the recruitment of phagocytic cells and the recognition, phagocytosis, and digestion of ACs. In these processes, signaling molecules could be divided into four types: find-me, eat-me, bridging, and don’t eat-me signals (Figure 1). First, the find-me signals are exhibited in different forms, including nucleotides, proteins, lipids, and lipid products.^5^ The best known types are the nucleotides, such as adenosine triphosphate (ATP) and uridine triphosphate (UTP). Released by ACs through pannexin 1, ATP/UTP can act as short-range chemoattractants to promote purinoreceptor-2-dependent recruitment of phagocytes.^6^ Other important contributors are proteins, such as fractalkine (CX3CL1), which is secreted from apoptotic lymphocytes and binds to its receptor (CX3CR1) on the surface of phagocytes to attract macrophages via caspase- and Bcl-2-regulated signals.^7^ In addition, the lipid mediator, lysophosphatidylcholine, and the lipid product, sphingosine-1-phosphate (S1P), contribute to the recruitment of macrophages and the inhibition of necrosis.^8^ Particularly, the lysophosphatidylcholine and G protein-coupled receptor G2A system can attract phagocytes to ACs and prevent autoimmunity in a “keep calm” mechanism.^9^ Second, eat-me signals are mainly composed of molecules on the surface of ACs, of which phosphatidylserine (PtdSer) is the most well-researched molecule. Eat-me signals also consist of modified molecules, such as intercellular adhesion molecule 3 and CD31. Normally, PtdSer is confined to the inner surface of the plasma membrane by the action of flippases that translocate PtdSer from the outer leaflet. When cells undergo apoptosis, PtdSer translocates to the cell surface due to the activation of scramblases and the inactivation of flippases.^10^ Later, the PtdSers on ACs are attached by PtdSer receptors on phagocytes, including T cell immunoglobulin mucin receptor 4 (TIM4) and TIM1, stabilins, and adhesion G protein-coupled receptor B1. ACs are engulfed and progress to the next stage. Third, bridging molecules are able to bind to both the ACs and the phagocytes with their receptor-binding domains. For instance, the molecules associated with PtdSer include growth arrest-specific 6 (GAS6), protein S (PROS1), and milk fat globule-epidermal growth factor 8 (MFG-E8),^5^^,^^11^ as well as the TAM (TYRO3, AXL, MERTK) receptors and the αvβ3 and αvβ5 integrins.^12^ Specifically, MFG-E8 acts as a bridge linking ACs and macrophages via PtdSer and the αβ/β integrins to facilitate the tethering step, and then triggers a series of downstream signals to clear ACs.^3^^,^^6^ Fourth, don’t eat-me signals usually exist on non-ACs, which prevents viable cells from being cleared by phagocytes. The best known don’t eat-me signal is CD47. Although CD47 can protect normal cells, it renders malignant cells resistant to efferocytosis and promotes tumor progression, suggesting its contradictory role in efferocytosis.

In conclusion, when apoptosis occurs, dying cells announce their presence to nearby phagocytes with find-me signals and then recruit motile phagocytes to the place of death. Meanwhile, ACs mark their outer leaflets with eat-me signals to allow themselves to be recognized by phagocytes. Eat-me signals are usually retained on the inner leaflets of the plasma membrane of healthy cells. Subsequently, phagocytes upregulate the corresponding cell surface receptors and bridging molecules to complete direct and indirect binding between the ACs and phagocytes.^13^ After combination, the Crk II/Elmo-Dock180-Rac pathway is activated to promote cytoskeletal reorganization of phagocytes, leading to corpse internalization.^14^^,^^15^ As a consequence (Figure 1), ACs are decomposed within the phagocytic cells by phagolysosomes. After digestion within the phagolysosome, cytokines are released, which further affects disease development (for details about molecules, see the reviews by Morioka et al.^3^ and by Gheibi Hayat et al.^5^). Since efferocytosis involves the signals and corresponding cytokines mentioned above, proper regulation of the signals may enhance AC clearance, and drugs targeting special molecules may provide novel approaches to treat disorders. However, whether the effect of intact or impaired efferocytosis is beneficial for the body depends on the diseases, which is discussed as follows.

### Overview of Efferocytosis upon Non-tumor and Tumor Diseases

Recently, a large number of studies have identified the role of efferocytosis in non-tumor and tumor diseases. The former primarily include atherosclerosis, lung diseases, and wounds, but the latter include breast cancer, prostate cancer (PCa), and leukemia. This section focuses on the relationships of efferocytosis and those diseases. The next section concentrates on the association between released cytokines during efferocytosis and these mentioned disorders.

#### Non-tumor Diseases and Efferocytosis

Although precise mechanisms relevant to efferocytosis among prevalent diseases are vague, researchers depict a relatively positive relationship between AC clearance and atherosclerosis, lung injury, and wound healing.

#### Atherosclerosis and Efferocytosis

When exposed to oxidized low-density lipoproteins (oxLDLs), there is a reduction of nitric oxide bioavailability, leading to endothelial activation and monocyte chemotaxis. This process then causes an accumulation of oxLDLs and differentiated macrophages, which generate an inflammatory stimulus within the subendothelial layer of arteries. Subsequently, excessive cholesterol accumulation induces transformation of lipid-laden phagocytes into foam cells, which eventually undergo apoptosis and necroptosis.^16^^,^^17^ Later, dying cells express eat-me molecules on their surface, such as calreticulin^18^ and PtdSer. These molecules interact with the receptors (e.g., integrin αvβ5, MERTK, transglutaminase 2, low-density lipoprotein receptor-related protein 1 [LRP1], and scavenger receptor B) on phagocytes via bridging molecules, resulting in the activation of enzymes involved in phagolysosomal degradation and efferocytosis during early lesion formation.^19^, ^20^, ^21^, ^22^ Efferocytosis also limits the progression of atherosclerosis via indirectly inhibiting the generation of reactive oxygen species (ROS) and pro-inflammatory mediators, and directly enhancing anti-inflammatory and anti-oxidant responses.^23^^,^^24^ Although efferocytosis functions in early stages of atherosclerosis, its capabilities begin to wane in advanced plaques, and the necrotic core occurs after overproduction of secondarily necrotic cells.^25^ In advanced plaques, the expressions of eat-me molecules and bridging molecules are decreased. However, increased tumor necrosis factor α (TNF-α) induces expression of the don’t eat-me molecule CD47 via nuclear factor κB (NF-κB), ultimately rendering vascular cells resistant to efferocytosis.^26^ Moreover, Doran et al.^12^ drew a comprehensive and detailed relationship between advanced plaques and defective efferocytosis.

One mechanism is linked to impaired efferocytosis in advanced atherosclerosis via the proteolytic cleavage of the key receptors, MERTK and LRP1, which increase plaque necrosis. Ait-Oufella et al.^27^ observed an accumulation of ACs and enhanced lesion development in low-density lipoprotein receptor-deficient female mice with MERTK deficiency after a high-fat diet. This is supported by Thorp et al.^28^ who found that mutation of the phagocytic MERTK receptor inhibits efferocytosis and accelerates the formation of necrotic plaques in Apoe^−/−^ mice. Other researchers have suggested that protease disintegrin and metalloproteinase domain-containing protein 17 (ADAM17) mediates the proteolysis of MERTK. Furthermore, the cleaved extracellular fragment named soluble MER can bind to GAS6, and thus suppress efferocytosis.^29^ Third, LRP1 may impair efferocytosis via exposure of macrophages to oxLDLs, or degradation by epsin-mediated and ubiquitin-dependent internalization.^30^ The fourth mechanism is associated with arachidonic acid-derived 12(*S*)-hydroxyeicosatetraenoic acid (12(*S*)-HETE), which suppresses ACs internalization via Ras homolog gene family, member A activation.^31^ Finally, CD47 is upregulated in ACs and allows them to avoid recognition by phagocytes.^12^

**Figure 2:**
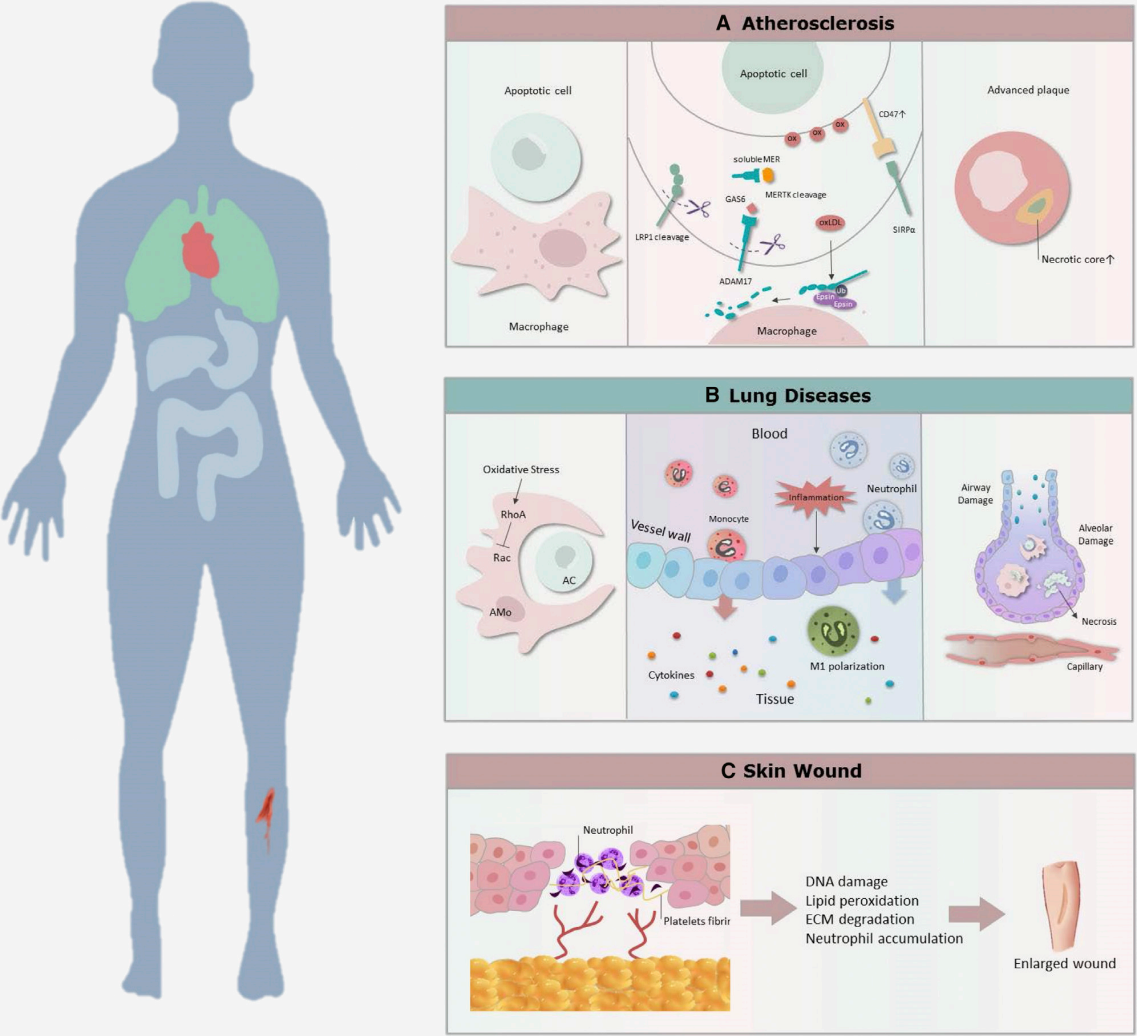
Roles and Effects of Impaired Efferocytosis in the Most Often Studied Non-tumor Diseases (A) In atherosclerosis, the impaired efferocytosis because of exposure to oxLDLs and disturbed processes of recognition, such as MERTK cleavage and CD47 upregulation, leads to the formation of necrotic and plaque development. (B) In lung diseases, the inefficient efferocytosis leads to damaged airway and cell necrosis due to an accumulation of neutrophils and released cytokines. As there are different types of phagocytic cells, results of efferocytosis vary among specific lung diseases. (C) During wound healing, the defective efferocytosis cannot clear ACs promptly, resulting in an enlarged wound.

Taken together, complete efferocytosis inhibits the development of atherosclerosis in the early stages, but efferocytosis is impaired in advanced stages (Figure 2A). The defective efferocytosis not only damages blood vessels, but it accelerates plaque necrosis through various mechanisms, such as MERTK cleavage and CD47 upregulation. These results indicate that improved efferocytosis and reduced oxLDLs in the early stages may significantly alleviate atherosclerosis.

#### Lung Diseases and Efferocytosis

Although the process of efferocytosis shares many similarities with atherosclerosis, lung diseases have a closer relationship with efferocytosis. This is due to the greater number of phagocytic cell types (alveolar macrophages, lung DCs, epithelial cells, and fibroblasts) and disease types (asthma, cystic fibrosis, acute lung injury [ALI], and chronic obstructive pulmonary disease [COPD]) involved in this process besides the complex inflammatory and immune responses. Within AC clearance, alveolar macrophages possess multiple AC recognition receptors, such as TAM receptors and αvβ3 integrin.^32^^,^^33^ CD103^+^ DCs have a role in acquiring and transporting ACs to the draining local lymph nodes, and in presenting antigens to CD8 T cells.^34^ Cultured human lung epithelial cells are reported to ingest apoptotic eosinophils.^35^ Additionally, with released molecules, such as vascular endothelial growth factor (VEGF), effective efferocytosis promotes the maintenance of normal airway and alveolar structures even in inflamed lung tissue.^36^^,^^37^

In contrast, defective efferocytosis increases the numbers of uncleared ACs, contributing to the development of several lung diseases, especially chronic disorders. For example, Aziz et al.^38^ found that MFG-E8^−/−^ mice exhibited lung damage because of neutrophilic infiltration and production of TNF-α and myeloperoxidase in LPS-induced ALI.^39^ Grabiec et al.^40^ indicated that deficiency of AXl receptor tyrosine kinase, which is highly expressed on mouse airway macrophages, exaggerates lung inflammation with extensive ACs in the airways in severe asthma. Also, alveolar macrophages from COPD patients have defective efferocytosis, especially in active smokers, and this defect is potentially linked to the S1P system, particularly S1P receptor 5.^41^^,^^42^ In addition, oxidant stress and proteolytic events can amplify the alveolar destruction and aggravate lung damage.^43^

To summarize, efferocytosis plays a significant role in maintaining lung homeostasis, and defective efferocytosis can cause acute and chronic lung injury due to extensive inflammation (Figure 2B). Nevertheless, the mechanism by which the phagocytic cells work together to have the utmost efficiency remains unknown. Furthermore, enhancers of efferocytosis, which could benefit patients with combined lung diseases, also remain unelucidated. These warrant thorough investigation to determine the extent of their potential in clinical use.

#### Wounds and Efferocytosis

Similar to the role of efferocytosis in atherosclerosis and lung diseases, efficient clearance of ACs and reduced inflammation contribute to wound healing. Generally, wound healing involves an intricate interplay between the immune system and phagocytic cells, which include four phases: hemostasis, inflammation, proliferation, and remodeling. During wound healing, there is an infiltration of abundant neutrophils. In addition, increased pro-inflammatory cytokines activate endothelial cells and platelets to exert protective effects.^44^ Of note, the timely clearance of apoptotic neutrophils is quite important for allowing wound healing to enter into the proliferative phase from the inflammation phase.^45^^,^^46^

However, when efferocytosis fails due to mitochondrial dysfunction or reduced stimulants, it will cause an overproduction of inflammatory mediators, including degranulation and ROS.^47^, ^48^, ^49^ Many studies demonstrate that the prolonged presence of neutrophils and associated mediators in the wound milieu promotes the formation of chronic wounds. For example, neutrophil-derived proteases, such as elastase and matrix metalloproteinases,^50^ can degrade healthy extracellular matrix. Additionally, neutrophil-induced ROS can aggravate destruction of the extracellular matrix. These events trigger additional production of inflammatory mediators (e.g., interleukin-1β [IL-1β], TNF-α) and proteolytic enzymes, promoting inflammation amplification.^51^^,^^52^

Therefore, efficient neutrophil recruitment is important for wound healing, and prompt efferocytosis assists in accelerating wound repair (Figure 2C). The most substantial aspect that warrants investigation is the time window of efferocytotic enhancement, especially in the second stage of wound healing.

#### Tumors and Efferocytosis

Recently, TAM receptors have been shown to be overexpressed by cancer cells, but also expressed by virtually all cells of the TME.^53^ Particularly within the TME, there lies an abundant cell type, known as tumor-associated macrophages.^54^ Upon those macrophages, the TAM receptors bind to PtdSer on ACs using Gas6 and PROS1 as bridging ligands to induce efferocytosis. However, they differ from non-cancer diseases, as these macrophages are further polarized to a pro-tumor M2-like phenotype, followed by an increased secretion of immunosuppressive cytokines, resulting in tumor progression.^55^ From this perspective, TAM receptor-directed therapies in preclinical studies and clinical trials may have anticancer effects (details can be found in tables 1 and 2 in the review by Myers et al.^56^). Although efferocytosis-related mechanisms describing internal relationships between tumor progression and efferocytosis are currently understudied, we review articles about several tumors with a high global burden, such as breast cancer, PCa, and leukemia to show the strong connection between these tumors and efferocytosis.

#### Breast Cancer and Efferocytosis

Using MDA-MB-231 breast cancer cells, Ma et al.^57^ found that the SIAH2-NRF1 (nuclear respiratory factor 1) axis regulates tumor mitochondrial function, tumor-associated macrophage polarization, and cell death to facilitate tumor maintenance by remodeling the TME. They also indicated that when efferocytosis is impaired by dampening the degradation of NRF1 under hypoxia, the tumor cells become susceptible to apoptosis in a FADD (Fas-associated protein with death domain)-dependent fashion and undergo secondary necrosis because of inhibited macrophage polarization. Moreover, widespread cell death triggers efferocytosis-induced wound-healing cytokines, such as IL-4, IL-10, IL-13, and transforming growth factor β (TGF-β), in the TME to further promote metastatic tumor progression.^58^^,^^59^ In this case, intact efferocytosis enhances tumor progression, while defective efferocytosis promotes tumor apoptosis and causes secondary necrosis, both of which are detrimental to the host. Likewise, Werfel et al.^60^ pointed out that in lapatinib-treated HER2^+^ mammary tumors in MMTV-Neu mice, efferocytosis cleared apoptotic tumor cells, while blockade of efferocytosis induced secondary necrosis of ACs. However, the combined inhibition of MERTK and indoleamine-2,3-dioxegenase (IDO1) blocked tumor metastasis and caused tumor regression in 60% of MMTV-Neu mice. These results suggest that apoptotic and necrotic tumor cells can exhibit their effects on tumors via efferocytosis and IDO1, respectively. Therefore, inhibition of MERTK and IDO1 produces an anti-tumor effect, which benefits the host.^60^^,^^61^

**Figure 3:**
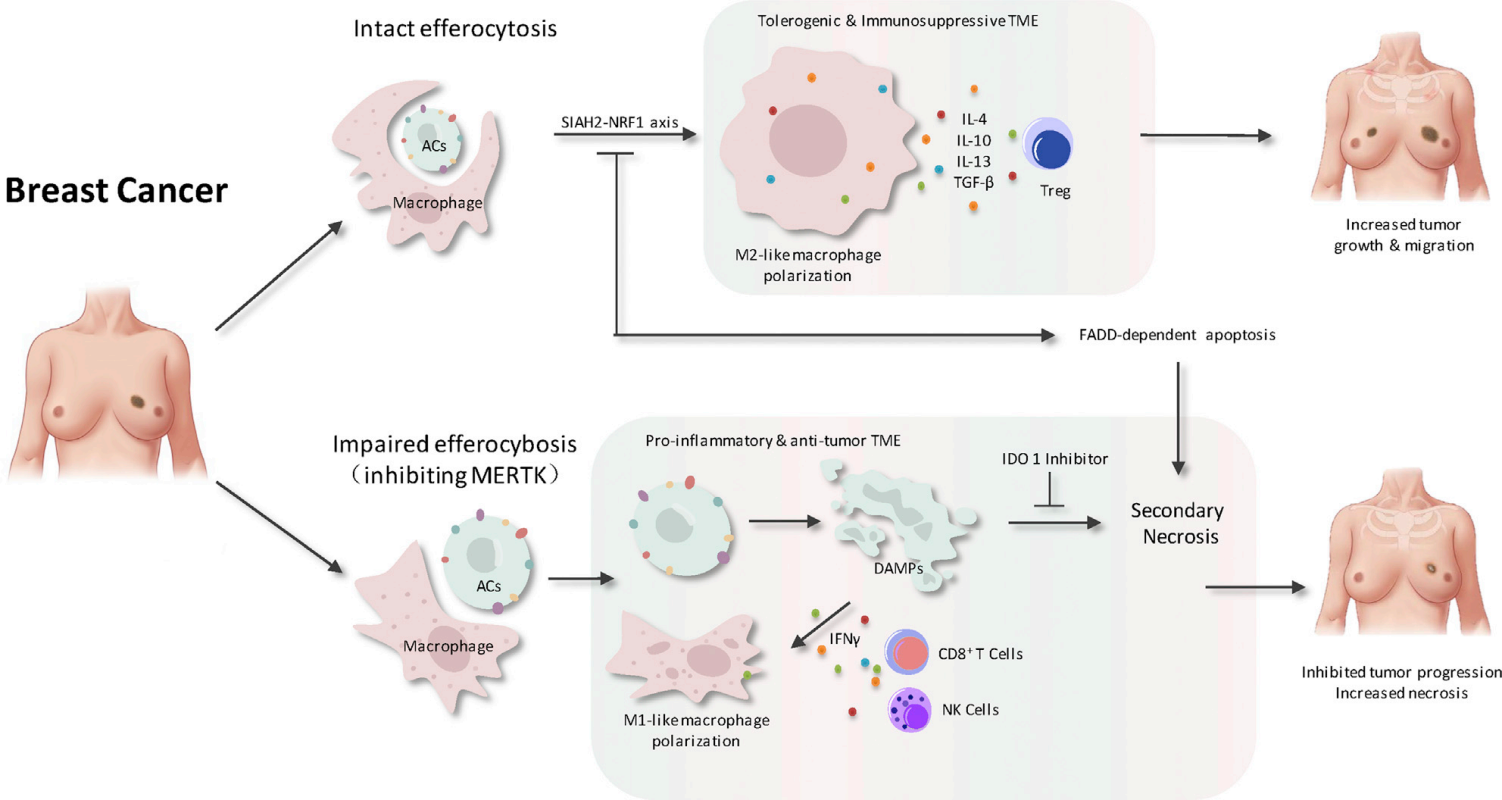
The Dual Function of Intact and Impaired Efferocytosis in Breast Cancer Efficient efferocytosis is stimulated by the SIAH2-NRF1 axis to trigger M2-like macrophage polarization and cytokine (e.g., IL-10, TGF-β) release to create a tolerogenic TME, leading to increased tumor progression and migration. Alternatively, impairing efferocytosis through inhibiting MERTK or NRF1 can lead to secondary necrosis and result in an unfavorable impact in hosts. However, co-inhibition of efferocytosis and necrosis via the MERTK inhibitor and IDO1 inhibitor can suppress tumor progression.

Both complete and impaired efferocytosis may have an unfavorable impact on hosts with breast cancer (Figure 3); however, combinations with other drugs may help to solve the adverse effects of efferocytosis inhibition (secondary necrosis). Thus, the combined treatment may suppress tumor progression and contribute to recovery of the host following chemotherapy, providing an innovative method of cancer treatment.

#### PCa and Efferocytosis

Numerous studies have confirmed the role of macrophages in promoting tumor progression in PCa. Jones et al.^62^ identified an increased level of M2-like macrophages *in vitro* during efferocytosis of apoptotic tumor cells compared with the M1-like macrophages, with increased numbers of PC-3 PCa *in vitro*. Also, Soki et al.^63^ suggested that MFG-E8-mediated efferocytosis can induce polarization of PCa-associated macrophages through the STAT3/SOCS3 pathway. In regard to PCa with bone metastasis, macrophage-driven efferocytosis of PCa cells *in vitro* induced the expression of pro-inflammatory cytokines, especially CXCL5, and the proinflammatory environment fueled further cancer cell growth. This mechanism is also confirmed by Roca et al.,^64^ who found that there were increased CXCL5 serum levels and more active efferocytotic activities existing in patients with PCa bone metastasis. Since efferocytosis accelerates tumor development, research aiming at impairing efferocytosis may suppress tumor progression. For example, administration of Stat3 reduces skeletal metastatic tumor growth and decreases skeletal tumor size, and CXCL5-deficient mice inhibit tumor progression, providing a clue for designing additional successful therapies.^62^^,^^64^

**Figure 4:**
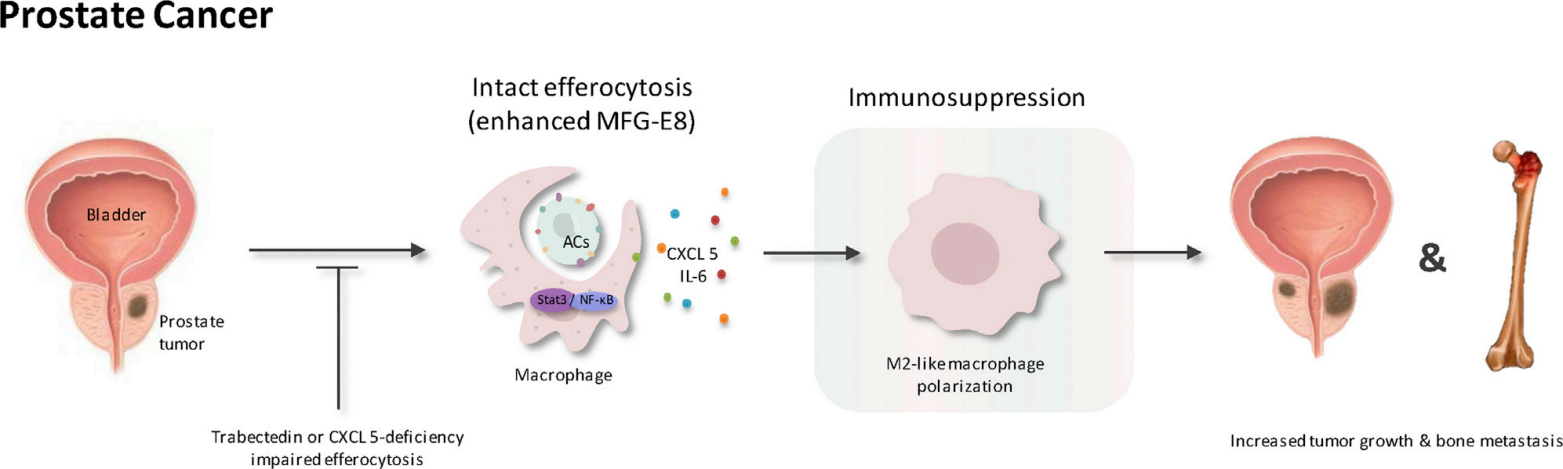
The Role of Intact and Impaired Efferocytosis in Prostate Cancer MFG-E8-mediated efferocytosis can induce M2 polarization of tumor-associated macrophages through the STAT3/SOCS3 pathway. Additionally, cytokines CXCL5 and IL-6 enhance M2 polarization, leading to tumor growth and bone metastasis of prostate cancer. Impairing efferocytosis by trabectedin administration or CXCL5 deficiency can suppress tumor size and bone metastasis.

Therefore, in metastatic cancers, affecting tumor-associated macrophage polarization and TME via efferocytosis may limit the progression of local and metastatic lesions (Figure 4). Future research should be focused on produced cytokines, as they have a very complex influence in disease.

#### Leukemia and Efferocytosis

During the process of efferocytosis, MERTK and AXL have been implicated as promoters of tumor cell survival in many hematopoietic malignancies, including acute leukemia, chronic leukemia, and multiple myeloma. TAM receptors can also facilitate leukemic phenotypes by interacting with other oncogenic proteins such as fms-related tyrosine kinase 3 (FLT3), LCK/YES-related novel protein tyrosine kinase (LYN), and fibroblast growth factor receptor 3 (FGFR3).^65^ In particular, acute myeloid leukemia (AML) is a Gas6-dependent cancer, and AXL/GAS6 expression predicts poor prognosis in AML.^66^, ^67^, ^68^ As an example, AXL is activated in the presence of GAS6 in AML cell lines. This activation triggers downstream signaling through the AKT/phosphatidylinositol 3-kinase (PI3K) and mitogen-activated protein kinase (MAPK) pathways, contributing to oncogenic transformation.^66^ Moreover, AXL physically interacts with proteins, such as FLT3, FGFR, and TYRO3, to accelerate tumor cell migration and invasion.^69^ In contrast, combined treatment with subtherapeutic doses of doxorubicin and an AXL inhibitor, BGB324, decreased tumor growth in an AML xenograft model. Targeting the TAM receptors and inhibiting the bone marrow microenvironment via impaired efferocytosis may be an effective treatment in leukemia.^69^

Currently, there are plentiful experimental studies regarding GAS6- and TAM-targeted treatments, which have been confirmed to reduce tumor progression *in vitro*. However, more *in vivo* studies and clinical research are still necessary.^70^ Moreover, further studies are still needed to uncover methods of severing connections between efferocytosis-related molecules and other proteins derived from mutated genes, which may promote tumor development.

#### Other Tumors and Efferocytosis

Although there are few studies that analyze the underlying mechanisms of efferocytosis in tumors, Wu et al.^70^ summarized that GAS6 promotes tumor progression in various tumors and systems (except intestinal tumor), including the circulatory system, locomotor system, gastrointestinal system, nervous system, and urinary system.^70^^,^^71^ Wu et al.^72^ suggested that treatment with MERTK suppression and fractionated radiation has a therapeutic effect in glioblastoma. Ishaque et al.^73^ demonstrated that several mutated non-coding elements have dual protagonists in metastasis and efferocytosis-/PD-L1 mediated immunosuppression in colorectal cancer. Other researchers have indicated that non-professional phagocytes require the recognition of PtdSer by receptor brain-specific angiogenesis inhibitor 1 and actin cytoskeleton remodeling to engulf subcellular fragments in human papillomavirus-positive cervical cancer.^72^, ^73^, ^74^ Further mechanistic studies and potential targeting are warranted.

### Interactive Roles of Cytokines in Efferocytosis in Non-tumor and Tumor Diseases

Cytokines are small molecule-soluble polypeptide proteins secreted by immune cells and tissue cells. They play a mutual regulatory role among cells to regulate cell growth, differentiation, and immune responses. Biological agents that target cytokines have valuable clinical application in the treatment of tumors, immune deficiency, and infection. Cytokines include six categories: interleukin, colony-stimulating factor (CSF), interferon (IFN), TNF, growth factor (GF), and chemokines. Since cytokines have certain functional characteristics, including pleiotropism, redundancy, synergy, antagonism, and network, the interactions between cytokines are very complex. It has been observed that cytokines released during efferocytosis may exert different effects on different disorders, making efferocytosis appear contradictory. Herein, we review the types of cytokines related to efferocytosis in the abovementioned six diseases and summarize the main functions of cytokines in terms of pro- and anti-inflammatory, as well as immunosuppressive, functions for further study.

#### Interleukin

A growing number of studies have shown several interleukins participating in efferocytosis and disease regulation. We review the most frequently detected interleukins, including IL-10, IL-1β, and IL-6.^75^, ^76^, ^77^

#### IL-10

Researchers have concluded that in atherosclerosis, interleukins such as IL-4, IL-13, and IL-10 activate specific transcription factors (e.g., STAT3 and STAT6, IFN regulatory factor 3, peroxisome proliferator-activated receptor-γ [PPARγ]) in macrophages to encourage inflammation resolution.^78^ This result has also been confirmed by many previous studies reviewed by Hopkins.^79^ In recent years, Proto et al.^80^ indicated that regulatory T (Treg) cell expansion could improve efferocytosis in advanced atherosclerosis through a transcellular signaling pathway. Treg cells secrete IL-13 to stimulate IL-10 production in macrophages, and autocrine-paracrine signaling by IL-10 induced Vav1 in macrophages to activate Rac1 and improve AC engulfment. Additionally, Manega et al.^31^ found higher 12(*S*)-HETE serum levels and lower IL-10 levels in patients with coronary artery disease than in healthy individuals, but the 12(*S*)-HETE-mediated inflammatory effect can be counteracted by atorvastatin (0.1 μM). Therefore, IL-10 plays a role in the reduction of inflammation in atherosclerosis. Additionally, recent strategies of Treg cell enhancement or atorvastatin administration may benefit people with advanced plaques, although more studies and clinical trials are needed.

Likewise, IL-10 has similar anti-inflammatory effects and shows improvement in most lung diseases,^80^ whereas it promotes infection in *Klebsiella pneumoniae*. Prolonged STAT6 activation and additional administration of ACs with simvastatin can contribute to the resolution of bleomycin-induced lung inflammation and fibrosis.^81^^,^^82^ Alternatively, the A54970 strain of *K. pneumoniae* can inhibit inflammasome activation and cell death by inducing IL-10 production, which will enhance bacterial survival and dissemination within the host.^83^ These contradictory effects of IL-10 in lung diseases reveal a different perspective toward efferocytosis in infectious diseases. In wound healing accompanied with increased IL-10, efferocytosis upregulated cellular fatty acids to fuel mitochondrial respiration and activate an NAD^+^-dependent signal transduction cascade to induce an anti-inflammatory response. This anti-inflammatory reaction is also found in another study, which implies that elevated macrophage miR-21 promotes efferocytosis and silences target genes PTEN and PDCD4.^49^^,^^84^

With regard to tumors, there are few studies directly showing the role of IL-10. Widespread cell death in breast cancer during postpartum involution triggers wound-healing cytokines, including IL-4, IL-13, and IL-10, in the TME to promote metastatic tumor progression. Other researchers found upregulation of antiphagocytic genes, such as IL-10 and IL-18, in African American patient-derived PCa TME.^58^^,^^85^ Whether IL-10 has crucial functions in anti-inflammation or in tumor progression similar to that of non-tumor diseases, and whether it is useful in inhibiting tumor development via reducing IL-10 in the TME, deserves future explorations.

#### IL-1β and IL-6

The role of IL-1β and IL-6 seems contrary to that of IL-10. Accompanied by enhanced levels of IL-1β and IL-6, a deficiency of macrophage scavenger receptor class B type I (SR-BI) and macrophage LRP-1 increases cell death and inflammation in atherosclerosis. The mechanism involves impaired efferocytosis via the Src/PI3K/Rac1 pathway, as well as inhibited Akt activation.^86^^,^^87^ Similarly, a reduced efferocytotic ability of alveolar macrophage to ACs, with increased IL-1β, encourages neutrophilic inflammation in bronchiectasis and asthma.^88^^,^^89^ Of note, one study indicated that IL-6 attenuates the macrophage pro-inflammatory phenotype and constitutes a protective response by eliminating excess cholesterol in human macrophages and phagocytes. This is realized by triggering the ATP-binding cassette transporter A1-mediated cellular free cholesterol efflux. Accordingly, a certain cytokine, IL-6, may play different roles via various ways.^90^ Although IL-1β is found to promote inflammation in non-tumor diseases, there is little evidence that elucidates the role of IL-1β in tumors or TME.

#### GFs

GFs refer to a group of cytokines that can promote the growth and differentiation of corresponding cells. TGF-β is the most well-researched GF, which plays a role in anti-inflammation, similar to the role of IL-10 in efferocytosis, and how VEGF sometimes participates in resolving wound inflammation and improving angiogenesis.^45^ In studies, M2 macrophages are found to secrete IL-10 and TGF-β to enhance atherosclerosis regression. Two main signaling pathways, the Akt/mTORC/LXR pathway and the JAK/STAT6 pathway, are associated with M2 polarization.^86^^,^^91^ Similarly, TGF-β has an anti-inflammatory function in lung diseases and wounds. For instance, PPARγ activation can resolve lung inflammation and fibrosis, but inhibition of PPARγ reversed these results by inhibiting efferocytosis and decreasing the expression of TGF-β and IL-10.^92^ Additionally, TGF-β signaling can induce alternative macrophage activation, and defective TβRII signaling downregulates interactions with other immune cells, such as T cells, by inhibiting M2 polarization. It is TGF-β that results in attenuated anti-inflammation in the lung.^93^ Nevertheless, efferocytosis with increased TGF-β and IL-10 secretion increased *Mycobacterium leprae* survival in macrophage-1 cells from paucibacillary leprosy patients, and it contributed to the persistence and sustained infection of the mycobacterium.^94^ Taken together, TGF-β contributes to macrophage polarization and inflammation attenuation in non-tumor diseases. However, as there is always an accompanying changed level of IL-10, whether this protection is derived from IL-10 or from TGF-β alone remains unclear. Whether it is effective to treat diseases by inducing macrophage polarization through increasing TGF-β levels also remains to be determined.

In tumors, TGF-β may encourage tumor development, and it has an unfavorable impact on the host. In oral tongue squamous cell carcinoma, HSC-3 cells induced the expression of epidermal growth factor and TGF-β in co-cultures with M2 macrophages. Direct cell-cell contact between them stimulates tumor cell migration and invasion.^95^ In postpartum tumors, there is increased TGF-β in the clearance of dying tumor cells, but TGF-β blockade inhibited postpartum tumor metastasis.^58^ Therefore, TGF-β acts as tumor contributor. However, studies are too few to draw this conclusion. The method by which TGF-β promotes tumor progression is not well described in studies. Finally, is this function associated with anti-inflammation in TME?

#### Colony-Stimulating Factor

CSF is a group of factors that can stimulate the differentiation and proliferation of pluripotent hematopoietic stem cells and hematopoietic progenitor cells at different developmental stages. CSF mainly includes granulocyte-macrophage colony-stimulating factor (GM-CSF), macrophage CSF (M-CSF), and erythropoietin (EPO). In some experiments, they have been observed in the process of efferocytosis. Since macrophages have been extensively used as a model for efferocytosis and polarization, Ivanov et al.^96^ revealed that mesothelial cells produce membrane-bound and secreted CSF1 to sustain peritoneal macrophage growth in order to maintain tissue homeostasis. Kawano et al.^10^ found that CSF treatments increased the percentage of CD62L senescent neutrophils in the spleen of null mice, to prevent mice from developing a lupus-like autoimmune disease. Other researchers observed that the delivery of recombinant GM-CSF to the alveolar space restored the efferocytotic capability of alveolar macrophages to protect against pneumonia. Lai et al.^97^ concluded that murine bone marrow-derived macrophages induced by M-CSF can be further polarized into the M2 phenotype by baicalin, to have an anti-inflammatory effect.^10^^,^^97^, ^98^, ^99^ In addition, EPO is involved in this process. Luo et al.^100^ indicated that PPARγ was significantly decreased in obese mice and EPO increased macrophage PPARγ to promote efferocytosis in obese mice. Moreover, EPO seems more suitable than PPARγ agonists in obesity-related inflammation regulation. The reason is that pre-adipocytes expressed EPOR, but EPO can downregulate PPARγ expression and inhibit pre-adipocyte differentiation *in vitro* and *in vivo*.^101^

In summary, CSF promotes tissue repair through inducing macrophages. However, leukocyte depletion frequently occurs in patients with tumors, and GM-CSF is often used to stimulate leukocyte growth during chemotherapy. Further clinical research is necessary to determine whether GM-CSF enhances tumor progression through inspiring efferocytosis, and how to control the adverse effects caused by GM-CSF.

#### IFN

IFN is named for its interference with viral replication, and it has antiviral, anti-cell proliferation, anti-tumor, and immune regulation effects. IFN is divided into type I and type II IFN. Type I IFN includes IFN-α and IFN and is produced by virus-infected cells. However, type II IFN, IFN-γ, is mainly produced by activated T cells and natural killer (NK) cells. Studies have proven that IFN is involved in efferocytosis. It was observed that IFN-γ-induced autophagy triggered the extracellular secretion of annexin A2 (ANXA2) in lung epithelial cells, leading to enhanced efferocytosis.^102^ Other studies have suggested a positive role of IFN to host. They identify IFN-β as an effector cytokine, involved in resolving bacterial inflammation, and they indicate that IFN-γ improved the responsiveness of macrophages to *Staphylococcus aureus*.^103^^,^^104^

IFN also functions in tumors, showing an anti-tumor role of IFN-γ. Long-term-activated DCs fail to effectively activate alloreactive IFN-γ-responsive T cells, and they changed their profile toward a non-functional and tumor-promoting phenotype.^105^ In addition, in the last step of efferocytosis, Cunha et al.^106^ showed that tumor-associated macrophages with LC3-associated phagocytosis (LAP) displayed M2 characteristics and reduced secretion of IFN-γ, which suppressed the anti-tumor response. TAM lacking LAP exhibited M1 characteristics and increased stimulator of IFN genes (STING)-dependent type I IFN production to promote IFN-γ, resulting in anti-tumor responses. It was the impairment of LC3-associated phagocytosis in myeloid cells of the TME that had anti-tumor effects. Although their studies support a role for LAP, these components possibly function in another biological process that does not involve phagocytosis. Since LAP interference may “reprogram” the function of tumor-related macrophages to facilitate the clearance of cancer cells without interfering with typical autophagy processes, targeting LAP-specific proteins may be a potential therapeutic strategy. Furthermore, one study utilizing TAM-IFN-γR1 reporter lines puts forward a rationale that PtdSer-targeting, anti-TAM receptor, and anti-PD-L1-based therapeutics will have great advantages.^107^

#### TNF

TNF was originally named for its ability to cause necrosis of tumor tissue, and there are two main types in the TNF family: TNF-α and TNF-β. TNF-α is produced by activated monocytes or macrophages, and TNF-β is mainly produced by activated T cells. The TNF family has a crucial role in regulating immune responses, killing target cells, and inducing apoptosis. We mainly review the function of released TNF in diseases, especially in tumors, to find potential therapeutic ways associated with efferocytosis. In atherosclerosis, mechanistic studies identify TNF-α as a fundamental driver of impaired AC removal, accounting for the activation of NF-κB and a compromised role of defective efferocytosis in vascular disease.^26^^,^^79^^,^^86^ However, the selective activation of cannabinoid type 2 receptor (CB2) by JWH-133 and HU-308, with reduced levels of TNF-α and oxLDL-induced ROS, improves efferocytosis of normal-cultured and oxLDL-loaded macrophages. This process promotes the stability of atherosclerotic plaques.^108^ The function of TNF-α is similar in wound healing because the successful efferocytosis of ACs can suppress LPS-induced NF-κB activation and TNF-α expression.^84^ However, there is a dual role of reduced TNF-α in lung diseases. On the one hand, it was observed that the CD73-A2a axis in macrophages causes reduction of TNF-α, linking early inflammatory events to subsequent immune responses to ACs.^109^ On the other hand, the reduced inflammatory response by fluticasone plus ACs through reducing TNF-α, CCL3, and CCL5 can increase the risk for community-acquired pneumonia (CAP) in COPD patients.^110^ In a sense, reducing TNF-α and suppressing inflammation is not always beneficial, and their effects vary among diseases.

As for tumors, a lack of experimental evidence hinders progress in learning the underlying relationship between TNF and efferocytosis, although there are hundreds of thousands of papers that illustrate the function of the TNF family in numerous tumors. For example, TNF-α is an essential pro-inflammatory cytokine found in the TME of breast cancer patients. TNF-α is involved in all stages of breast cancer progression, including cell proliferation, survival motility, and acquired resistance to chemotherapy. TNF-α also mediated pro-tumorigenic pathways through binding to TNFR1 and TNFR2. As a consequence, tumor proliferation and migration are enhanced via several signaling pathways, such as the p42/p48 MAPK, PI3K/AKT, and p38/MAPK pathways, in different breast cancer cell lines.^111^ It is necessary to further study the association between TNF and efferocytosis in tumors to find effective potential targets, especially in inhibiting tumor metastasis.

#### Chemokines

Chemokines are cytokines that initiate chemotaxis in different cells, including the C subfamily, CC subfamily, CXC subfamily, and CX3C subfamily. They not only attract and activate immune cells, but they also participate in regulating blood cell development, angiogenesis, and apoptosis. They are also involved in many pathological processes, such as tumorigenesis, metastasis, and pathogenic microbial infection. In atherosclerosis, many studies have supported the role of the chemokine receptor CCR5 and its ligands CCL3, CCL4, and CCL5 in triggering the progression of atherosclerosis, particularly in later stages of plaque. There was a reduction of more than 50% in the size of plaque lesions in the aortic root and the abdominal aorta of Apoe^−/−^Ccr5^−/−^ mice.^112^, ^113^, ^114^, ^115^ Since it may be easier to detect mRNA levels of the ligands CCL3, CCL4, and CCL5 in circulating leukocytes than in chemokine receptors, the ligands could serve as clinically relevant biomarkers.^115^

Recently, the role of chemokines in the lungs has drawn interest among researchers. Different from the effect in atherosclerosis, genetic deletion of the CCL2 receptor in mice with β1 integrin-deficient type 2 alveolar epithelial cells (AECs) reduced AEC efferocytosis and accelerated inflammation. This study indicates a necessary requirement for recruited monocytes/macrophages in limiting lung injury.^116^ However, COPD macrophages release high levels of chemokines, including CXCL8 and CCL2, to recruit inflammatory cells to the lungs, promoting disease progression.^43^ Nevertheless, fluticasone plus ACs reduced CCL3, CCL5, and CXCL1, leading to an increased risk for CAP in COPD patients with bacterial infection.^110^ Taken together with the result that the monocyte chemoattractant protein-1 (MCP-1)/CCL2 improved efferocytosis in murine bacterial pneumonia to resolve acute lung inflammation, chemokines that attract inflammation cells have a dual function.^117^ It encouraged inflammatory responses to clear bacteria and prevented further injury to the lung, and it promoted tissue repair via enhanced efferocytosis. Alternatively, it induces inflammation and impairs efferocytosis, preventing effective clearance of excessive inflammatory cells. There is a dynamic balance of inflammatory response and a “flexible” effect of chemokines and efferocytosis. Therefore, whether the function of chemokines and efferocytosis is good or bad for the host varies depending upon various situations. In wound healing, responses of a number of CXC and CX3C chemokines temporarily recruit neutrophils to injured sites during the inflammatory phase, and they accelerate the clearance of necrotic neutrophils during the transition into the proliferative phase, supporting a positive role of chemokines in the wound.^44^

In addition to non-tumor diseases, chemokines also have a position in tumors. Researchers have observed that macrophage-driven efferocytosis of PCa *in vitro* activated Stat3 and NF-κB (p65) signaling to stimulate the expression of the pro-inflammatory cytokine CXCL5, whose deficiency reduced tumor progression. CXCL5 mediated inflammation and tumor growth in bone, it may serve as a potential target for cancer therapeutics.^64^ Akalu et al.^118^ indicated that chemokines (e.g., CCL2, CCL3, CCL4, CCL5, CXCL9, and CXCL10) allow effector T cells to migrate into the TME to mediate tumor destruction, but poor chemokine-mediated trafficking of T cells favors tumor growth.^119^, ^120^, ^121^ Lacking evidence, the source of released chemokines during efferocytosis in tumors remains unclear. Whether there are other functions related to different chemokines in the TME should be studied further. In addition, is it possible to use chemokines to predict survival rate in patients with tumors?

#### Summary of Cytokines Associated with Efferocytosis in Diseases

As mentioned above, cytokines play an important role in diseases via efferocytosis, although the underlying mechanisms differ depending on the cytokine types and disorders, and several cytokines have dual functions, depending on the situation. According to numerous studies on atherosclerosis, lung diseases, and on tumors, we conclude that IL-10, TGF-β, and IFN-γ contribute to the resolution of inflammation, and that CSF enhances tissue repair. IL-1β, TNF-α, and certain chemokines promote inflammation in non-tumor diseases. In tumors, IL-4, IL-13, IL-10, TGF-β, and CXCL5 increase tumor cell growth and metastasis through efferocytosis-induced cell clearance and macrophage polarization in the TME. IFN-γ and other cytokines, such as CCL2, CCL23, CXCL9, and CXCL10 have anti-tumor roles. There is a dynamic balance of efferocytosis related to inflammation and tissue repair or tumor progression. Finding effective targets related to cytokines, such as CXCL5 and IL-1β, may shed light on the above-mentioned diseases.

Regardless of the small amount of evidence showing TNF-α and efferocytosis in tumors, TNF-α has important implications in the course of various diseases. For instance, studies have exhibited a dual role of TNF-α in treating breast cancer as a drug and a target. In animal models, both TNF-α intra-tumoral administration, in combination with different conventional therapies, and application of TNF-α monoclonal antibodies decrease tumor growth and metastatic potential of breast tumors.^111^ Potential therapy through TNF-α occurs in other solid tumors, such as PCa, in which the miR-130b/TNF-α/NF-κB/VEGFA loop suppresses PCa angiogenesis.^122^ In leukemia, TNF-α contributes to tumorigenesis and activates the suppressive functions of Treg cells. These improve the tumor’s ability to evade the immune system, indicating a way to use anti-TNF therapy in leukemia.^123^ Although the clinical usage of TNF-α has been limited by toxicity and side effects, using TNF-α alone or in combination with chemotherapy and radiotherapy as an adjuvant in cancer may offer great promise for tumor treatment. Furthermore, employing nanoparticles or tumor-homing peptides may be a more viable option.^124^

### Conclusion and Perspectives: Will Efferocytosis Be the Future Direction?

In the previous sections, we reviewed the processes and signaling molecules of efferocytosis, including the roles of efferocytosis and associated cytokines in non-tumor diseases (especially well-studied atherosclerosis, lung diseases, and wound healing), solid tumors (especially breast cancer and prostate cancer), and leukemia. With the combined information of recent articles and reviews, we find a paradoxical effect of efferocytosis in non-tumor and tumor diseases and some notable points of each type.

In atherosclerosis, effective efferocytosis in the early stages contributes to inflammation resolution, but in the advanced stage, reducing oxLDLs and inhibiting cleavage of MERTK can suppress the impairment of efferocytosis. Additionally, increasing IL-10, IL-4, IL-13, and TGF-β and reducing IL-1β, Il-6, TNF-α, CCL3, CCL4, and CCL5 can slow the progression of atherosclerosis. Of note, some miRs participate in the early and later stages of atherosclerosis by modulating endothelial activation, monocyte chemotaxis, and by regulating foam cell accumulation and inflammatory responses, respectively. These miRs may represent potential molecular targets for anti-atherosclerotic therapy, though the selectivity of miR-based therapy delivery systems remains a major challenge in drug development (see figure 2 and table 1 in Tajbakhsh et al.^75^). The main function of intact and defective efferocytosis is similar in inflammation reduction and tissue repair in lung diseases. However, there may be an adverse effect in bacterial pneumonia because of the decreased inflammation, as a result of improved efferocytosis, and cannot clear infected cells, but CCL2 can enhance efferocytosis in murine pneumonia to promote lung repair, suggesting a dual function of efferocytosis in certain lung diseases.^110^^,^^117^ Moreover, there are various macrophages exhibiting functions in different lung diseases, but the role of efferocytosis is dependent on the situation. As for wounds, valid efferocytosis and its cytokines have a positive role in healing, similar to its function in atherosclerosis and lung diseases, but improving the rate of AC clearance is a key factor, as there is an inflammatory phase during the repair process several days after injury.^47^ In conclusion, apart from some opposite results in several conditions, efferocytosis has benefits to hosts with non-tumor conditions.

Although intact efferocytosis shows anti-inflammation and immune suppression in tumors, especially the TME, it creates an environment suitable for tumor cell growth and migration. Efferocytosis induces conversion of tumor-associated macrophage to the M2 phenotype, which inhibits the clearance of ACs and promotes the expression of CD47 to escape from recognition.^58^^,^^125^ Impairing efferocytosis to block the above mechanisms seems possible. Additionally, targeting the TAM receptors and inhibiting the bone marrow microenvironment may be useful in ameliorating leukemia.^69^ Nevertheless, inhibiting efferocytosis may not provide substantial benefits for the body because of the effects caused by secondary necrosis, but suppressing efferocytosis and necrosis via inhibition of MerTK and IDO1 may exhibit an anti-tumor effect without damage to the host in breast cancer patients.^60^^,^^61^ Furthermore, as cytokines can assist tumor progression, targeting IL-4, IL-13, IL-10, TGF-β, and CXCL5 or enhancing IFN-γ and some chemokines (e.g., CCL2, CCL3, CXCL9, CXCL10) may inhibit tumor invasion and metastasis. A combination of chemotherapy or radiotherapy may provide a novel therapeutic method for cancer treatment. The toxicity and efficacy of those drugs are under consideration. However, there is an interesting point related to efferocytosis and anticancer drugs. When anticancer drugs cause apoptosis-induced death of cancer cells, efferocytosis in TME is started, followed by excessive anti-inflammatory cytokines (TGF-β, prostaglandin E_2_ [PGE2], platelet-activating factor [PAF], and IL-10). This process leads a silent anti-tumor response by the immune system. Activated efferocytosis facilitates tumor progression, making the situation quite complicated. Drugs such as doxorubicin may promote the expression of eat-me signals on the surface of a tumor. Therefore, a serious reconsideration is expected on anticancer drugs and efferocytosis in treating tumors.^5^

For future studies, we propose four questions related to efferocytosis and hope to provide an effective path for drug development. First, how can researchers efficiently delay the progress of atherosclerosis and how can they effectively control the time window or neutrophil recruitment to maximize the effects of efferocytosis in tissue repair or wound healing? Second, how can one make full use of TAM receptors to mitigate illnesses, since TAM receptors have a crucial role in efferocytosis? Third, what methods can be used to polarize involved macrophages into the necessary phenotype to modulate anti-inflammation and the immune system? Fourth, what can be done to combine efferocytosis and chemotherapy to treat tumors without subjecting the host to any potential toxic effects? As there are few studies describing the relationship between tumors and efferocytosis, more research is needed.

## Author Contributions

A.S., D.L., X.K., and S.T. drafted the manuscript; L.S. revised the manuscript; Y.C., X.W., and A.S. reviewed and modified the manuscript. All authors agreed on the final version.

## Conflicts of Interest

The authors declare no competing interests.
